# Collaboration between general hospitals and community health services in the care of suicide attempters in Norway: a longitudinal study

**DOI:** 10.1186/1744-859X-9-26

**Published:** 2010-06-11

**Authors:** Erlend Mork, Lars Mehlum, Elin Anita Fadum, Ingeborg Rossow

**Affiliations:** 1 National Centre for Suicide Research and Prevention, Institute of Clinical Medicine, University of Oslo, Norway; 2 Norwegian Institute for Alcohol and Drug Research, Oslo, Norway

## Abstract

**Background:**

The aim of this paper was to study the collaboration between emergency departments (EDs) in general hospitals and community health services (CHS) in Norway when providing psychosocial care and aftercare to patients treated in EDs following a suicide attempt. We wanted to explore the extent to which quality indicators at the hospital level measured in 1999 and 2006 could predict the presence or absence of a chain of care structure in the CHS in 2006.

**Methods:**

Data were collected through structured interviews with informants from 95% of all general hospitals in Norway in 1999 and 2006, and informants from CHS, in a stratified sample of Norwegian municipalities in 2006 (n = 47).

**Results:**

In 15 of the 47 municipalities (32%), the CHS reported having a chain of care structure in 2006. A discriminant function analysis revealed that the hospitals that in 1999 had: (a) a collaboration agreement with aftercare providers, and (b) written guidelines, including a quality assurance system, were significantly more likely to have municipalities with a chain of care structure in their catchment area in 2006.

**Conclusions:**

Hospitals' and municipalities' self-reported provision of aftercare services for patients treated after a suicide attempt was markedly below the recommendations given in national standards. Systems at the hospital level for the management and care of patients admitted after a suicide attempt and systematic collaboration between hospitals and aftercare providers seem to be important elements in the long-term maintenance of continuity of care for suicide attempters.

## Background

It is well established that patients admitted for attempted suicide or other kinds of deliberate self-harm have a significantly elevated risk of repetition and completion of suicidal behaviours, especially in the first year after discharge [1-4]. Among the few strategies that bear evidence of effectiveness in reducing this risk is a chain of care for these patients [5,6]. A chain of care implies significant challenges, both with respect to delivering services to patients with multiple and complex problems and with respect to organising and coordinating various actors and care providers in services that are funded and organised differently and on different care levels. Many of these patients leave hospital without adequate suicide risk assessment after a suicide attempt [7,8] and they often do not attend or drop out of aftercare treatment [9]. Although many experts in the mental healthcare field consider continuity of care and integrated care important, the research literature is weak with regard to the effectiveness of such interventions. A meta-analysis of studies on the effectiveness of community mental health teams found a clinically, but not statistically, significant reduction in deaths from suicide [10]. Even if we draw on research literature from a broader area, such as psychiatric services or integrated care, irrespective of patient group, there is still little available systematic knowledge that could inform us about what a chain of care structure should contain, how the service should be organised and delivered and how sustainable it will be over time [11]. The present study was designed to expand our knowledge on the factors at the hospital level that seem to be of particular importance for the maintenance of a chain of care structure at the municipality level by analysing longitudinal data on chain of care structures for suicide attempters in Norway.

In Norway, a main objective of the National Strategy for Suicide Prevention (1994) was to improve the quality and continuity of care for patients admitted to general hospitals following a suicide attempt [12]. Supported by time limited governmental funding, about 30% of Norwegian general hospitals with an Emergency Department (ED)-implemented chain of care programmes for suicide attempt patients in the period from 1995 to 1999. Nearly all of the health services in Norway are publicly funded and these services are organised at 2 levels; hospital services are organised at the regional level (comprising 4 health regions with 55 general hospitals), and primary healthcare services (community health services) are provided at the municipality level (comprising 431 municipalities). This implies that a chain of care presupposes collaboration between a hospital and each of the many municipalities within the hospital's catchment area.

Based on experiences with the chain of care model and international recommendations [13-16], national quality standards for the clinical management of patients admitted to general hospitals after a suicide attempt were issued in 2001 by the Norwegian Board of Health [17]. General hospitals with an ED were required to satisfy quality standards regarding suicide risk assessment, training of staff, coordination of monitoring and management of suicide attempting patients and procedures for systematic collaboration with aftercare providers (see Table 1 and [18] for details). The national standards do not clearly define what constitutes a chain of care within community health services (CHS) at the municipality level, but recommend that CHS has a team or a coordinator responsible for receiving referrals and interacting with the local hospital in rapid (within 1 week from discharge) and active aftercare. Follow-up usually consists of consultations, aimed at crisis intervention, with the individual and their family, pending other treatment to be established, and guidance and support in establishing or maintaining contact with other community based services (for example, health and social services, school) and/or specialist mental health services.

**Table 1:** Quality indicators in the treatment of patients admitted following a suicide attempt

Quality of care indicator	1999				2006				Stability (quality of care indicator present both in 1999 and 2006)			
	**CCS in CHS in 2006 (n = 15), n (%)**	**Not CCS in CHS in 2006 (n = 32), n (%)**	**Analysis**		**CCS in CHS in 2006 (n = 15), n (%)**	**Not CCS in CHS in 2006 (n = 32), n (%)**	**Analysis**		**CCS in CHS in 2006 (n = 15), n (%)**	**Not CCS in CHS in 2006(n = 32), n (%)**	**Analysis**	
									
			**χ**^**2**^**(df)**	***P*value**			**χ**^**2**^**(df)**	***P*value**			**χ**^**2**^**(df)**	***P*value**

01: ED has a monitoring system	6 (40)	13(41)	0.00 (1)	0.97	11(73)	10 (31)	7.32	0.007*	6 (40)	6 (19)	2.43	0.119
02: ED has a team or a coordinator	13 (87)	16 (50)	5.81 (1)	0.02	13 (87)	15 (47)	6.71	0.010	13 (87)	9 (28)	14.06	0.000*
03: ED has written guidelines, including a quality assurance system	9 (60)	6 (19)	8.00 (1)	0.005*	11 (74)	17(53)	1.73	0.188	7 (47)	4 (13)	6.65	0.010*
04: ED has provided training of staff	13 (87)	15 (47)	6.71 (1)	0.010*	13 (87)	16 (50)	5.81	0.016	12 (80)	6 (19)	16.21	0.000*
05: ED provides systematic supervision of staff	6 (40)	3 (9)	6.19 (1)	0.013	8 (53%)	15 (47)	0.17	0.680	6 (40)	2 (6)	8.24	0.004*
06: ED has structured collaboration with aftercare provider in CHS	13 (87)	12 (38)	9.92 (1)	0.002*	15 (100)	14 (44%)	13.68	0.000*	13 (87)	6 (19)	19.56	0.000*
07: ED staff routinely make suicide risk assessment	15 (100)	32 (100)		NS	13 (87)	31 (97)	1.78	0.182	13 (87)	31 (97)	1.78	0.182
08: ED has specific procedure for patients who have not been assessed for suicide risk	9 (60)	16 (50)	0.41 (1)	0.52	9 (60)	19 (59)	0.00	0.968	7 (47)	12 (38)	0.36	0.551
09: ED has specific guidelines for follow-up care	11 (73)	22 (69)	0.10 (1)	0.75	15 (100)	23 (72)	5.22	0.022	11 (73)	17 (53)	1.73	0.188
10: ED refers at least 90% of patients^a^	9 (60)	18 (56)			6 (40)	26 (84)			4 (29)	16 (53)		
11: Patients receive information about available help resources after discharge	13 (87)	18 (56)	4.21 (1)	0.04	14 (93)	18 (57)	6.46	0.011	13 (87)	11 (34)	11.18	0.001*
12: ED establishes contact with aftercare provider within first work day after discharge^a^	9 (60)	25^a^(78) (miss = 5)			15 (100)	30 (97)	0.50	0.482	9 (60)	24 (89) (miss = 5)		

Data reported for Emergency Departments (EDs) in 1999, 2006 and in both 1999 and 2006 (stability) based on whether the community health services (CHS) did or did not have a chain of care structure (CCS) in 2006.

*Difference is significant based on an α level of *P *< 0.05 with Bonferroni correction for multiple testing (10 tests) and correction for the correlation between the quality of care indicators: 1999: *P *< 0.012, 2006: *P *< 0.009, Stability: *P *< 0.011

^a^These quality of care indicators had missing observations (range = 1-5) and were excluded from further analysis.

df = degrees of freedom; NS = not significant.

In three previous studies [18-20] we have reported on assessments of the quality of hospital services provided for patients admitted after a suicide attempt. The results indicate that maintaining high-quality clinical services to suicide attempt patients seems dependent on clearly defined standards and routines described in written guidelines, a system to ensure that the guidelines are followed and training of staff with respect to clinical management and care of patients. These and other previous studies have been limited to the standards and services given at EDs in general hospitals and we have no systematic knowledge about the aftercare services provided in the CHS. In 2006, we therefore conducted an interview study of the CHS in a sample of randomly selected municipalities in Norway. The purposes of the present paper are: (a) to assess how common it was for Norwegian CHS to have a chain of care structure for suicide attempters in 2006, (b) to explore the capacity of quality indicators measured in 1999 and 2006 at the general hospital EDs to predict the presence of a chain of care structure in the CHS in 2006, and (c) to explore whether hospitals that had implemented a time-limited chain of care programme between 1995 and 1999 were more likely to have municipalities with a chain of care structure in their catchment area in 2006.

## Methods

### CHS in municipalities in 2006

#### Participants and procedures

Data were collected through interviews with key persons in municipalities in Norway in 2006. A randomly selected sample of 50 municipalities and a substitution sample of 50 municipalities, stratified into 4 geographic/administrative regions and 3 levels of urbanisation, were drawn from the population of all 431 Norwegian municipalities by Statistics Norway (Oslo, Norway). Municipalities with a population of fewer than 3,000 inhabitants, which account for 6.1% of the Norwegian population, were excluded. The selected municipalities were sent a written invitation to participate in the study. The invitation was specifically addressed to the employee within the CHS in the municipality who was most involved in the care of patients referred after a suicide attempt. The interviews were conducted by telephone and lasted about 30 min. Of the original 50 municipalities, 46 agreed to participate (92%); thus, 4 additional municipalities were drawn from the substitution sample. Two additional municipalities were substituted, one due to intermunicipality cooperation with another participating municipality and the other because the informant became unavailable due to illness. In total, six substitutions were made. Of the 50 municipalities, 47 (94%) were served by a general hospital ED that had participated in both the 1999 and 2006 ED survey [18,20] and the analyses reported in this paper are based on information from these 47 municipalities, covering close to one-third of the entire Norwegian population.

#### Measures

The interview addressed structural and organisational aspects of the psychosocial follow-up of patients referred to CHS in the municipality following a suicide attempt. The structured interview protocol was based on the protocol developed for the 1999 ED survey, with changes made for the purposes of the current study and after having conducted pilot interviews. One member of the project group (EAF) conducted all of the CHS interviews between June and December 2006. The outcome measure was whether or not the CHS in the municipality had a chain of care structure (CCS) based on the following criteria: (a) the CHS had a person or unit responsible for the psychosocial follow-up of patients after hospital treatment for a suicide attempt, (b) the CHS had structured cooperation with the local hospital concerning the psychosocial follow-up of patients hospitalised for a suicide attempt, and (c) the CHS ensured that, at discharge, hospitals provide the patient with information about aftercare providers in the CHS.

Data on the number of inhabitants in each municipality and the degree of urbanisation of the municipality (rural, rural/urban or urban) were gathered from Statistics Norway. Information on the position of the informant in the CHS (leader/non-leader) was gathered through the interview.

### EDs at Norwegian general hospitals in 1999 and 2006 Participants and procedures

Data were collected through interviews with key persons in EDs in general hospitals in Norway in 1999 and 2006. All Norwegian general hospitals with EDs were invited to participate and at the interview we specifically targeted the employee who was most involved in the care of patients admitted following a suicide attempt. The response rates were 95% at both time points (55 of 58 hospitals in 1999 and 52 of 55 hospitals in 2006). A total of 31 of the hospitals provided services to the 47 participating municipalities described above. The interviews were conducted by telephone and lasted about 30 min.

For details on the materials and methods of the hospital study, see Mork *et al.*[20,21].

### Measures

Interviews focused on 12 indicators of the quality level of the psychosocial care delivered to patients admitted to the EDs following a suicide attempt. These indicators were designed to reflect essential aspects of routines for the care and management of suicide attempters according to recommendations and guidelines published in the international literature [13-16,22-24]. Each of these 12 quality of care indicators, shown in Table 1, represents an element of the treatment the ED either had or had not, and were hence scored dichotomously. In a separate paper, we have described the nationwide results [18]. In the present study, only data on those EDs (n = 31) corresponding to the CHS in the 47 municipalities are presented. Due to missing observations on quality of care indicators 10 and 12 in both 1999 and 2006 (range = 1-5), these variables were excluded from statistical analysis. The remaining 10 quality of care indicators in the EDs in 1999 and 2006 were explored for their ability to predict whether or not the CHS had a chain of care structure in 2006. Stability in the ED's quality of care was scored positively if the measured quality of care indicator was present at the ED at both time points. We acquired information on which hospitals implemented a chain of care programme funded by the National Plan for Suicide Prevention between 1995 and 1999 [12,25].

A rigorous definition of suicide attempt could not be applied in these surveys. However, it is our experience that in clinical practice in most inpatient EDs and CHS in Norway, the term 'suicide attempt' generally refers to self-harm behaviour with a varying degree of intention to die. Nevertheless, since all participating ED's were inpatient units, a fairly high medical lethality was generally present in the patients who belonged to the population treated in this context.

### Statistical analysis

The main research question, to what extent the EDs quality of care indicators could predict whether or not the CHS had a chain of care structure in 2006, was assessed by x^2 ^tests in bivariate analyses, and discriminant function analysis (DFA). DFA is a linear stepwise regression model to predict group membership from a set of predictors. We were interested in assessing which of the quality of care indicator variables in EDs at different times of measurement could best discriminate between CHS with or without a chain of care structure (Wilks' A). Multiple logistic regression analysis models of whether or not CHS had a chain of care structure were also estimated, to check the robustness of results from DFA. The statistical significance level in the bivariate analysis was set to *P *< 0.05 with Bonferroni correction for multiple tests (10) and correction for correlation between predictor variables (see Table 1). All analyses were conducted using SPSS v.14 (SPSS, Chicago, IL, USA).

## Results

### Chain of care structure in the CHS in 2006

Almost half of the CHS (47%) had a unit or person responsible for coordinating the psychosocial follow-up of suicide attempters after hospital discharge. Two-thirds (68%) of the CHS had structured collaboration with their local general hospital concerning the post-discharge psychosocial follow-up of suicide attempters, and 43% had regular meetings with the local hospital as a part of this collaboration. A total of 53% of CHS ensured that, at discharge, hospitals provided the patients with information about the aftercare providers in the CHS.

In 15 (32%) of the municipalities, CHS reported that in 2006 they had a chain of care structure (CCS) that filled the 3 criteria listed above. No significant differences were found between CHS with and without a CCS with respect to population size, degree of urbanisation or the position of the informant (leader/non-leader).

### Predictors of a chain of care structure in the CHS

To examine whether quality of care indicators at EDs in 1999 could predict whether or not the CHS had a chain of care structure in 2006, a series of analyses were conducted with the presence of a CCS in the CHS in 2006 as the dependent variable. First, all quality indicators present in 1999, 2006 and at both times of measurement were entered bivariately as presented in Table 1. Two quality indicators from the hospital survey in 1999 were significantly associated with the presence of a CCS in the CHS in 2006: EDs that had established a structured collaboration with aftercare providers and EDs with written guidelines, including a quality assurance system. Only two characteristics of the EDs in 2006 were significantly associated with the presence of a chain of care structure in the CHS the same year: EDs that had established a structured collaboration with aftercare providers and EDs that had a monitoring system. However, when looking at stability at the EDs over time (quality indicator present in both 1999 and 2006), six quality of care indicators were significantly associated with CCS in the CHS (see Table 1).

To explore the degree to which characteristics of EDs at different time points were able to correctly classify CHS with and without a chain of care structure in 2006, a discriminant function analysis was conducted, first with the 10 quality of care indicators as predictor variables (Table 2). A model that included EDs with structured collaboration with aftercare providers and a team or coordinator on both measurement points (giving stability over time) provided the highest percentage (87%) of correctly classified CHS with and without a chain of care structure. Including the variable 'chain of care programme during the late 1990s' in the analysis increased the correct classification in 1999 (79%) and 2006 (83%) but did not change the results on the stability measure. Similar results were obtained by multiple logistic regression analyses (data not shown).

**Table 2:** The relationship between quality indicators at emergency departments (EDs) and the presence of chain of care structure (CCS) at community health services (CHS) in 2006

Predictor variables at EDs	1999				2006				Quality of care indicator present both in 1999 and 2006 (stability)			
	**Standard canonical discriminant functions**	**CCS in CHS, % (n) correct classified**	**Not CCS in CHS, % (n) correct classified**	**Total, % (n) correct classified**	**Standardised canonical discriminant functions**	**CCS in CHS, % (n) correct classified**	**Not CCS in CHS, % (n) correct classified**	**Total, % (n) correct classified**	**Standardised canonical discriminant functions**	**CCS in CHS, % (n) correct classified**	**Not CCS in CHS, % (n) correct classified**	**Total, % (n) correct classified**

**Predictors entered: the 10 quality indicators (Table 1):**												
QI 6: ED has structured collaboration with aftercare providers	1.0	87 (13/15)	63 (20/32)	70 (33/47)	1.0	100 (15/15)	56 (18/32)	70 (33/47)	0.756			
QI 2: ED has a team or a coordinator									0.477	80 (12/15)	91 (29/32)	87 (41/47)

**Predictors entered: the 10 quality indicators + chain of care programme funded by the national strategy**												
QI 6: ED has structured collaboration with aftercare providers in CHS	0.626				0.766				0.756			
Chain of care programme funded by the National strategy	0.798				0.590	80 (12/15)	84 (27/32)	83 (39/47)				
QI 1: ED has a monitoring system	-0.587	73 (11/15)	81 (26/32)	79 (37/47)								
QI 2: ED has a team or a coordinator									0.477	80 (12/15)	91 (29/32)	87 (41/47)

Data from EDs reported for 1999, 2006 and for EDs where quality indicators were present in both 1999 and 2006 (stability). Discriminant function analysis.

### Impact of having implemented a chain of care programme in the 1990s

Of the 47 CHS, 22 (47%) were served by a local hospital that had participated in a chain of care programme funded by the National Plan for Suicide Prevention between 1995 and 1999. CHS with CCS were significantly more likely to belong to a hospital area that had participated in a chain of care programme in the 1990s than CHS that had not (with CCS: 80%, without CCS: 31% (x ^2 ^= 9.75, degrees of freedom (df) = 1, *P *= 0.002).

## Discussion

Although all municipalities in Norway are supposed to have a chain of care, only one-third of the CHS in this representative sample of municipalities reported that they in fact had such a structure. The observed discrepancy between recommendations given in national guidelines and the reality described is much in line with international research on general hospital services for suicide attempters. Studies from other countries have shown that a large proportion of self-harm patients attending accident and emergency departments do not receive a psychosocial assessment [7,26]. Furthermore, substantial shortcomings have been found in staff training, interdisciplinary working and service planning, and availability of all-hours specialist psychosocial assessment [27-29].

Several explanations could apply to the observed discrepancy between policy and reality; the number of suicide attempts treated in general hospitals each year may be low in small municipalities/rural areas and this may negatively influence their ability to maintain services and routines over time. On a more general level, factors such as organisational changes, reforms, downsizing, change of leadership or loss of key personnel may contribute to the difficulty of establishing and maintaining collaboration between different levels of service provision over time. The lack of interventions that show evidence of effectiveness in preventing suicide after a suicide attempt [30] may also explain suboptimal collaboration between ED's and CHS. However, several previous studies suggest that shortcomings in hospital services provided to suicide attempters are associated with an increased risk of repeated suicidal behaviour [26,31,32]. Furthermore, an ecological study by Cooper and colleagues [33] suggests that the availability of mental health resources (mental health treatment, crisis treatment, case management) in the county of residence is associated with a reduction of suicidal behaviour in the year following a suicide attempt.

Stability over time on quality indicators at the EDs was the best predictor of whether or not community healthcare services had a chain of care structure in 2006, both on the level of single quality indicators and in the multivariate analyses. This study highlights several factors that could potentially contribute to a chain of care structure in the CHS in the hospital's catchment area. Maintaining a structured collaboration with aftercare providers and having a team or a coordinator in the hospital over time were both indicators associated with a relatively high prediction of the presence or absence of a chain of care structure in CHS in a representative sample of municipalities. A significantly higher likelihood of having a chain of care structure was also observed for CHS collaborating with hospitals that had written guidelines with a quality assurance system, training and systematic supervision of staff and routinely gave patients information about available aftercare providers/services during discharge in both 1999 and 2006. One common denominator of these variables is that they require the hospital to allocate human resources to the maintenance and implementation of local guidelines and practices both within the hospital system and towards aftercare providers.

Having implemented a chain of care programme at general hospitals in 1995 to 1999 significantly predicted the presence of a CCS in CHS 7-10 years later. This finding is in line with the results from another study conducted at our centre: hospitals that had implemented a chain of care programme in the latter half of the 1990s had standards and routines more in accordance with the recommendations than hospitals without such programmes in both 1999 and 2006 [18]. The chain of care programmes were of limited duration and funding. Even though an observational study such as the present work cannot directly measure the effectiveness of a chain of care programme, it is nevertheless noteworthy to observe that this limited intervention was associated with higher standards of care at another level of service provision as much as a decade later.

## Strengths and limitations

The hospital study was conducted at two time points in a nationwide fashion with remarkably high response rates. The sample size in the CHS study was relatively small. However, in light of the selection procedure and the fact that the CHS in the municipalities actually covered one-third of the entire Norwegian general population, we believe our findings are reasonably generalisable to the CHS in other parts of Norway and may have relevance to CHS in many developed countries. Due to low statistical power, there could be other variables that are important but did not reach statistical significance. The results reported in this paper have considerable overlap with the findings from the longitudinal nationwide survey of hospitals in Norway in 1999 and 2006 [18], which supports the associations observed. The main limitations of this study lay inherent within the study design and the limited conclusions that can be drawn from an observational study based on self-reported data gathered from different levels of service provision. We do not know if the informants' accounts of the quality of care delivered to patients correspond with the patients' points of view. Nevertheless, we still believe this type of research may provide valuable information to advise policy makers and clinicians in their implementation and prioritisation of different types of intervention and routines for management and follow-up of suicide attempters.

This study has measured selected structural elements in the CHS as indicators of the presence of a chain of care for patients discharged from general hospitals following a suicide attempt. There are a number of other ways the chain of care model could be defined or operationalised. Selecting structural elements as indicators of a chain of care model is one way of managing the considerable heterogeneity in population size, population density and organisation and content of the services delivered across municipalities and service providers.

Is this what is needed to strengthen the continuity of care for persons with suicide attempts? Currently, we do not know what system of aftercare service provision may best serve this mixed patient group. It could be that the patients will be best served by anchoring the chain of care in the general practitioner/family doctor. However, it is not clear that all general practitioners will have the interest or ability to provide active follow-up of these patients.

## Conclusions

The provision of aftercare services for patients treated after a suicide attempt at the community level is markedly below the recommendations of the national standards in Norway. It is likely that this has a negative effect on the patients' prognosis. The results of this study suggest that interventions aimed at establishing and maintaining structured collaboration with aftercare providers and a team or coordinator at the hospital level might be important aspects in fostering the presence of a CCS in the CHS. Regular training and supervision of staff in the assessment and psychosocial care and aftercare of patients admitted to EDs of general hospitals following a suicide attempt, and establishing local guidelines with a system for quality assurance, also seem to be important factors in maintaining a chain of care model. More thorough studies addressing programme content, structural elements, theoretical underpinnings and the effects of different chain of care programmes are needed to get a further grasp of what works and for whom. This study represents a first step giving a bird's eye view.

## Implications for practice

In a national context, clinical standards and practices should be monitored and evaluated against national recommendations at regular intervals. Because most of the healthcare system in Norway and some other developed countries are publicly funded, the health authorities can and should take advantage of this fact in demanding structured collaboration between the different levels of service provision to strengthen the continuity of care.

## Competing interests

The authors declare that they have no competing interests.

## Authors' contributions

LM designed the study. EM, LM, EAF and IR wrote the protocol. EM drafted the first manuscript and performed the statistical analysis. EM and EAF participated in the acquisition of data. All authors participated in the interpretation of data, revised it critically for important intellectual content and have read and approved the final manuscript.
